# Glial Cells and Their Contribution to the Mechanisms of Action of Cannabidiol in Neuropsychiatric Disorders

**DOI:** 10.3389/fphar.2020.618065

**Published:** 2021-02-04

**Authors:** Franciele F. Scarante, Melissa A. Ribeiro, Ana F. Almeida-Santos, Francisco S. Guimarães, Alline C. Campos

**Affiliations:** ^1^ Department of Pharmacology, Ribeirão Preto Medical School, University of São Paulo, Ribeirão Preto, Brazil; ^2^ Department of Physiology and Biophysics, Biological Science Institute, Federal University of Minas Gerais, Belo Horizonte, Brazil

**Keywords:** cannabidiol, pharmacology, neuropsychiatric disorders, glial cells, neurons

## Abstract

Cannabidiol (CBD) is a phytocannabinoid with a broad-range of therapeutic potential in several conditions, including neurological (epilepsy, neurodegenerative diseases, traumatic and ischemic brain injuries) and psychiatric disorders (schizophrenia, addiction, major depressive disorder, and anxiety). The pharmacological mechanisms responsible for these effects are still unclear, and more than 60 potential molecular targets have been described. Regarding neuropsychiatric disorders, most studies investigating these mechanisms have focused on neuronal cells. However, glial cells (astrocytes, oligodendrocytes, microglia) also play a crucial role in keeping the homeostasis of the central nervous system. Changes in glial functions have been associated with neuropathological conditions, including those for which CBD is proposed to be useful. Mostly *in vitro* studies have indicated that CBD modulate the activation of proinflammatory pathways, energy metabolism, calcium homeostasis, and the proliferative rate of glial cells. Likewise, some of the molecular targets proposed for CBD actions are f expressed in glial cells, including pharmacological receptors such as CB1, CB2, PPAR-γ, and 5-HT1A. In the present review, we discuss the currently available evidence suggesting that part of the CBD effects are mediated by interference with glial cell function. We also propose additional studies that need to be performed to unveil the contribution of glial cells to CBD effects in neuropsychiatric disorders.

## Introduction

During the first decades of neuroscience and psychopharmacology research, glial cells and cannabidiol (CBD) did not play a major role in modifying brain functions. Currently, however, both CBD and glial cells, initially thought as secondary components, are recognized as major players in the central nervous system (CNS) physiology and *Cannabis sativa* pharmacology, respectively.

CBD was isolated in 1940 by Adams and Hunt (1940) and had its chemical structure described 23 years later by Mechoulam and Shvo (1963). In the early 1970s, CBD has been shown not to mimic the effects of Cannabis sp*.*, and some believed CBD was an innocuous compound (Mechoulam et al., 1970). Most of the initial studies on CBD’s actions aimed to investigate how it could interact and antagonize delta-9 tetrahydrocannabinol (THC) effects (Karniol et al., 1974; Hine et al., 1975; Brady and Balster, 1980; Zuardi et al., 1982). However, several groups worldwide have dedicated their efforts to characterizing CBD’s pharmacological properties and therapeutic applications, especially since the 1990s (Guimarães et al., 1990, 1994; Zuardi et al., 1993; Hampson et al., 1998; Moreira and Guimarães, 2005; Campos and Guimarães, 2008). These efforts produced evidence for CBD’s potential actions against different disorders and have sustained the foundation for current public health policies around the globe that approved CBD-based medicines to treat conditions such as glaucoma, epilepsy, and cancer-associated pain (Campos et al., 2016).

Glial cells were named for their supposedly sole function of “gluing” the CNS matrix for sustaining the neuronal environment (Andriezen, 1893; Taylor, 1897). Nowadays, this vision has been expanded to include far more complex actions of glial cells on several vital aspects of the CNS homeostasis’s maintenance (Allen and Barres, 2009; Valles et al., 2019; Verkhratsky et al., 2019; Salas et al., 2020).

Several pharmacological receptors used as drug targets to treat neurological and psychiatric conditions are expressed in glial cells. In the present review, we will address the pharmacological effects caused by CBD in these conditions and discuss how interaction with glial cell function could help to explain them.

## The Great Potential of CBD as an Alternative to Treat Neuropsychiatric Disorders

The wide range of its therapeutic potential, together with, until now, good safety profile (Campos et al., 2016), has made CBD special among the almost 150 phytocannabinoids that have been already described (Hanuš et al., 2016). CBD is potentially useful in several of the main disorders that affect the CNS, including epilepsy, schizophrenia, autism, addiction, traumatic and ischemic brain injury, multiple sclerosis (MS), and anxiety, depressive, post-traumatic stress, obsessive–compulsive, and neurodegenerative disorders (Campos et al., 2016). In this section, we will briefly discuss the main studies that have investigated the effects of CBD in the context of these disorders.

### Anticonvulsant Properties

The term epilepsy refers to a disorder of brain function characterized by a periodic and unpredictable occurrence of seizures due to hyper excitability and hyper synchrony of neurons (McNamara, 1994; Austin and Dunn, 2002; Dichter, 2009; Jacobs et al., 2009; Devinsky et al., 2013; Ali, 2018).

Izquierdo et al. (1973) and Carlini et al. (1973) were the first to report a potential therapeutic application for CBD in epilepsy by describing its action on diminishing seizures in rats. In the animal model of epileptic seizures induced by acute pilocarpine administration, CBD has reduced the percentage of rats experiencing severe scores of seizures (Jones et al., 2012; Patra et al., 2019). Additionally, other authors also demonstrate that intracerebroventricular injection of CBD during the significantly diminished seizure scores during the chronic phase (Hosseinzadeh et al., 2016). In another rodent model based on the administration of the GABA inhibitor, pentylenetetrazole (PTZ), CBD reduced seizure severity and lethality (Consroe et al., 1982; Jones et al., 2010; Patra et al., 2019; Lima et al., 2020). Moreover, Mao et al. (2015) demonstrated that CBD not only decreases the daily average grade of epileptic seizures, but also promoted reduction of neuronal loss due in the hippocampus (Mao et al., 2015).

Seizures can also occur after intoxication by the abuse of certain drugs, such as cocaine (Köppel et al., 1996; Gobira et al., 2015; Schifano et al., 2019). CBD is able to increase the latency and reduced the duration of cocaine-induced tonic seizures (Gobira et al., 2015; Vilela et al., 2015), as effect no mediate by CB_1_ or CB_2_ (Gobira et al., 2015). Conversely, in PTZ model, it is hypothesized that CB_1_ and CB_2_ receptors, primarily located in neurons are involved in the anti-seizure effects of CBD (Vilela et al., 2017).

The anti-seizure effects of CBD have been observed in several clinical studies (Porter and Jacobson, 2013; Lattanzi et al., 2018; Thiele et al., 2018; Silvestro et al., 2019). In infantile refractory epilepsy such as Lennox-Gastaut and Dravet syndrome, randomized controlled trials evaluated the efficacy of CBD oil as an adjuvant agent and the CBD addition significantly reduced the frequency of seizures compared to placebo (Devinsky et al., 2017; Devinsky et al., 2018).

### Autism Spectrum Disorders

Autism spectrum disorders (ASDs) are a group of disabilities characterized by repetitive patterns behaviors and diminished social interaction that starts during childhood (Lai et al., 2014; Baxter et al., 2015; Goel et al., 2018). Currently pharmacological treatment of one part of the symptoms of ASDs includes antidepressants, anxiolytics and atypical antipsychotics (Stachnik and Gabay, 2010; Wink et al., 2010; Hurwitz et al., 2012; Goel et al., 2018). Recently, some studies have suggested CBD as a therapeutic strategy for the treatment of ASDs (Földy et al., 2013; Barchel et al., 2019; Poleg et al., 2019).

In this regard, low doses of CBD increased time of social exploration with the stranger mice in the Three-Chamber Test, and reduced autistic-type social deficits in genetic mouse model of Dravet syndrome (Kaplan et al., 2017). Besides ASDs, other psychiatric comorbidities as hyperactivity, Attention Deficit Hyperactivity Disorder and self-mutilation have been reported in the Dravet syndrome (Sturm et al., 2004; Murray, 2010; Berkvens et al., 2015).

The pharmacotherapy of behavioral changes in children with ASDs commonly involves the use of psychostimulants such as methylphenidate, however, the consensus has been that psychostimulants promote minimal clinical improvement for this population and many case reports have suggested a high rate of significant adverse effects (Handen et al., 2000). In a recent study conducted on children with ASDs, CBD treatment improved hyperactivity in over 68.4% of children. Comparing the overall improvement in hyperactivity symptoms in children treated with CBD to that of children treated with methylphenidate treatment as reported by literature, non-inferiority of CBD was observed. However, in this study, the main adverse effects induced by CBD were somnolence and change in appetite that occurred in a transient way and resolved spontaneously. Still, no symptoms of toxicity were reported (Barchel et al., 2019). These initial findings point the promising therapeutic effects of CBD for ASD’s. However, the exact action mechanism remains largely unknown.

### Anxiolytic Properties

Anxiety disorders are highly prevalent psychiatric conditions commonly associated with a diminished sense of well-being and elevated rates of incapacity (Kroenke et al., 2007; Mata et al., 2015; Sjöberg et al., 2017). The treatment of these disorders is based on the use of benzodiazepines and antidepressants (serotonin reuptake inhibitors (SSRIs), serotonin–norepinephrine reuptake inhibitors (SNRIs), tricyclic antidepressant and partial 5-HT1A receptor agonists) as well as non-pharmacological treatments, such as psychotherapy and physical activity (Phillips, 2017; Marwood et al., 2018; Ribeiro et al., 2018; Kandola et al., 2019). Unfortunately, the late onset of therapeutic effects and important adverse reactions reduces adherence and success of the pharmacotherapy (Blessing et al., 2015; Pruckner and Holthoff-Detto, 2017; Artigas et al., 2018; Davies et al., 2019).

Several studies have investigated CBD as a possible tool for treating these disorders (Stern et al., 2012; Campos et al., 2013b; Shbiro et al., 2019). The first studies conducted in animals showed contradictory results. Low doses of CBD induced anxiolytic-like behaviors, while in high doses promoted an anxiogenic-like action (Zuardi and Karniol, 1983; Guimarães et al., 1990; Onaivi et al., 1990).

Using the elevated plus maze (EPM), a classic model for screening anti-anxiety drugs in rodents, Guimaraes et al. demonstrated that a single systemic administration of CBD promoted anxiolytic-like behavior in rats (Guimarães et al., 1990, 1994). The anxiolytic effect of CBD has been reported in other animal models such as the Vogel conflict test (VCT) (Moreira et al., 2006), open-field and in the light-dark test (Long et al., 2010). Chronic administration of CBD (14 days or 21 days) also produces anxiolytic-like effect in rodents previously exposed to chronic stress (Campos et al., 2013b).

In order to evaluate the possible neurobiology of the anxiolytic effects of CBD, several studies infused CBD into brain areas governing panic and anxiety (Fogaça et al., 2014; Lee et al., 2017). CBD injected into the dorsolateral periaqueductal gray (dlPAG) produced anxiolytic-like effects in the EPM and VCT. This effect was blocked by antagonism of 5HT1A receptors, but not by CB_1_ receptors antagonism (Campos and Guimarães, 2009). The same mechanism was also responsible for the anti-panic effect of CBD in animals submitted to the electrical stimulation model of the dorsal PAG or elevated T maze (Soares et al., 2010). Corroborating these findings, in another brain region that modulates anxiety behavior, the prefrontal cortex, CBD also promotes the anxiolytic and anti-stress effects (Fogaça et al., 2014). Anxiolytic-like effect probably occurs by altering prefrontal-subcortical connectivity through amygdala and cingulate cortex and, a reduction in the activity of para-hippocampal gyrus, hippocampus and inferior temporal gyrus (Fusar-Poli et al., 2010; Crippa et al., 2011).

Zuardi et al. (1993) conducted a study in which the effect of CBD (300 mg) was compared with placebo, diazepam (10 mg; benzodiazepine) and ipsapirone (5 mg; 5-HT1A partial agonist compound) in healthy volunteers submitted to a simulated public test (SPS). The anxiety promoted by SPS was mitigated by ipsapirone and CBD, without triggering significant adverse reactions, while the anxiolytic effects induced by diazepam were accompanied by sedation (Zuardi et al., 1993).

### Antidepressant-Like Effects

CBD also modulate depressant-like behaviors in rodents. Using the forced-swimming test (Porsolt et al., 1977; Cryan et al., 2002), it was observed that the administration of CBD induced an antidepressant-like effect (El-Alfy et al., 2010; Zanelati et al., 2010; Shbiro et al., 2019). The same results were found in other animal models of depression, such as tail suspension and olfactory bulbectomy (El-Alfy et al., 2010; Linge et al., 2016). Repeated administration of CBD (30 mg/kg) also induces antidepressant-like effects in swiss mice (Schiavon et al., 2016). Single doses of CBD can also induce long-term antidepressant effects, a ketamine-simile effect (Linge et al., 2016; Sales et al., 2019).

Recent studies have shown that the antidepressant effect promoted by the systemic administration of CBD in mice submitted to the forced swimming test is associated with increased expression of synaptophysin, PSD95 (synaptic plasticity marker) and BDNF levels in medial prefrontal cortex (PFC) (Sales et al., 2019). The similar effects were described in mice submitted previously to chronic mild stress mouse model (Xu et al., 2019). Indeed, preceding research shows that CBD injection into the ventral medial PFC also induces antidepressant like behavior (Sartim et al., 2016).

### Stress-Related Disorders

Stress-related disorders are psychiatric conditions that could appear after the exposure and one or several stressful situations. It includes obsessive compulsive disorder (OCD) (Thomas et al., 2009; Seibell and Hollander, 2014) and posttraumatic-stress disorders (PTSD) (Rauch et al., 2006). SSRIs are the first-line drugs for the treatment of OCD and PTSD, which suggests that 5-HT-mediated neurotransmission is involved in their pathophysiology (Zohar et al., 2000).

Casarotto et al. (2010) demonstrated that CBD (single or repeated doses) decrease defensive responses in the marble burying test. Another study using the metachlorophenylpyperazine (mCPP), a nonselective 5-HT1A/D and 5-HT2C receptors agonist (Kennett et al., 1989) showed that CBD pre-treatment reduced the number of buried marbles (Casarotto et al., 2010; Umathe et al., 2011; Nardo et al., 2014).

Regarding the putative effects of CBD on PTSD, a number of good studies are available in the literature. Using the fear conditioning paradigm, several groups showed that the administration of CBD in rodents reduced the expression of fear, interrupting the reconsolidation of memory and facilitating the process of extinction (Stern et al., 2012; Berardi et al., 2016; Jurkus et al., 2016; Song et al., 2016; Bitencourt and Takahashi, 2018). CBD also promoted contextual fear conditioning extinction when infused into the infra-limbic region of medial prefrontal cortex (Do Monte et al., 2013). In spontaneously hypertensive rats (SHR), the treatment with CBD mitigate acquisition of contextual fear memory (Levin et al., 2012).

Another animal model used to study some aspects of PTSD is based on prey *vs.* predator paradigm. The exposure of rats to the predator (cat) triggers a long-lasting anxiogenic behavior, symptoms found in patients with PTSD. Campos et al. (2013a) demonstrated that repeated administration of CBD prevents long-lasting anxiogenic effects promoted by a single predatory exposure followed by an upregulation of 5-HT1A mRNA in hippocampus and prefrontal cortex (Campos et al., 2012a). Similar effects CBD-induced were observed also when mice (prey) were exposed to a constricting snake (predator) (Uribe-Marĩo et al., 2012).

In humans, a case-report suggested the putative effects of CBD in PTSD (Shannon and Opila-Lehman, 2016). Recently, Elms et al. (2019) conducted a retrospective review of medical records of 11 adult psychiatric patients diagnosed with PTSD who consented to CBD treatment as a complement to their routine of psychiatric treatment (drugs + psychotherapy). CBD administration for 8 weeks decreased the severity of PTSD symptoms in 91%. Neuroimaging studies have shown that the CBD administration promoted a change in the activity of amygdala, thalamus, the anterior cingulate gyrus, ventromedial prefrontal cortex (vmPFC), important structures in modulating behavior in patients with diagnosis of PTSD (Lanius et al., 2003; Milad et al., 2007; Passie et al., 2012).

### Drug Addiction

Addiction is a chronic and recurrent psychiatric disorder characterized by complex behavioral and neurobiological features that promote the compulsive and non-controlled use of a particular drug, such as cocaine, alcohol and opioids (Camí and Farré, 2003; Volkow and Li, 2005; Viudez-Martínez et al., 2018). It constitutes a public health problem in several countries (Lhermitte et al., 2012; Modesto-Lowe et al., 2017) with few effective treatments available.

In this scenario, CBD has been investigated as a possible therapeutic strategy for the treatment of drug addiction (Hay et al., 2018; Luján et al., 2018). In the self-administration model (Sanchis-Segura and Spanagel, 2006; Panlilio and Goldberg, 2007) CBD attenuated the self-administration of methamphetamine, but not heroin, in rats (Ren et al., 2009; Hay et al., 2018). Mahmud et al. (2017) also noted that acute administration of CBD did not alter cocaine self-administration or cue-induced relapse to cocaine seeking. However, in a 7-days treatment regimen, CBD attenuated cue-induced reinstatement of cocaine self-administration in rats (Gonzalez-Cuevas et al., 2018). In the conditioned place preference (CPP) test (Tzschentke, 2007), CBD potentiated the extinction of both cocaine and amphetamine use (Parker et al., 2004; Luján et al., 2018).

Regarding ethanol, CBD promoted significant reduction of ethanol consumption following by decreased neuronal tyrosine hydroxylase gene expression in the ventral tegmental area and reduced neuronal GPR55 signaling in the nucleus accumbens (NAc) (Viudez-Martínez et al., 2018).

In humans, a double-blind placebo randomized clinical suggested that CBD treatment (during one week) reduced the total number of cigarettes smoked (Morgan et al., 2013). In addition, 10-week treatment with CBD improved psychological and cognitive symptomatology observed in an open-label clinical trial realized in 20 ongoing cannabis users (Solowij et al., 2018). In individuals in abstinence of heroin acute administration of CBD, in contrast to placebo, significantly reduced the crack and anxiety induced by the presentation of protruding drug signs compared to neutral signs (Hurd et al., 2019). These data reinforce the results obtained in animal studies, however, the mechanisms involved in these actions need to be clarified.

### Antipsychotic Properties

Schizophrenia is a complex disorder characterized by the presence of psychotic symptoms, such as delusions and hallucinations, and by a core of negative symptoms, social isolation and anhedonia, affecting about 1% of world’s population (Egan and Weinberger, 1997; Freedman, 2003; Nucifora et al., 2019).

Zuardi et al. (1991) were pioneers in CBD research with potential antipsychotic properties. In this study, using apomorphine-induced stereotypy in rats, CBD, similar to the antipsychotic haloperidol, decreased the stereotyped behavior (related to positive symptoms of schizophrenia) in a dose-related manner. Moreover, contrary to haloperidol, CBD did not induce catalepsy, even at high doses (Zuardi et al., 1991).

Supporting this idea, CBD reduced the hyperlocomotion induced by the administration of d-amphetamine, an indirect dopaminergic agonist (Moreira and Guimarães, 2005). CBD also attenuated the hyperlocomotion observed after the administration of NMDA-antagonist, ketamine in the open field test (Moreira and Guimarães, 2005; Gururajan et al., 2012).

In the pre-pulse inhibition (PPI) of the startle response test, acute treatment with CBD ameliorates startle reflex deficits in rats (Long et al., 2006; Zuardi et al., 2012). Recently, Pedrazzi et al. showed that the pre-treatment with CBD (systemic or intra-NAc) attenuated the disruptive effects of amphetamine in mice submitted to the PPI ((Pedrazzi et al., 2015).

CBD is also effective in chronic models of schizophrenia in rodents. CBD can reduce psychotic-like effects induces by the chronic treatment with NMDA receptor antagonists, such as MK801 (during 28 days), by restoring the performance of mice in the social interaction test (related to negative symptoms) and new object recognition test (NOR-evaluates memory) (Gomes et al., 2015; Rodrigues da Silva et al., 2020). CBD treatment for 6 days rescued cognitive deficits induced by ketamine in rats submitted to NOR by reducing the transcriptional changes induced by ketamine in prefrontal cortex (Kozela et al., 2020).

In humans, a randomized, double-blinded study showed that CBD treatment produced clinical improvement of some symptoms of schizophrenia that is accompanied by a significant increase in serum levels of anandamide (AEA), resulted from the inhibition of the fatty-acid amide hydrolase (FAAH), enzyme that metabolizes this endocannabinoid (Leweke et al., 2012). Additionality, in another study, schizophrenic patients received CBD or placebo along with their pre-existing antipsychotic medication for 6 weeks and it was observed that CBD reduced the negative symptoms of schizophrenia as well as improved the patients’ cognitive performances (McGuire et al., 2018).

### Neurodegenerative Diseases

Neurodegenerative diseases are severe and debilitating conditions produced by the progressive degeneration and death of neurons in the brain triggered by several factors such as inflammatory processes, reactive-oxygen species (ROS), cytotoxicity, mitochondrial and protein dysfunction (Lassmann et al., 2001; Tiraboschi et al., 2004; Almeida-Santos et al., 2017). CBD has antioxidant, anti-inflammatory, anti-apoptotic and neuroprotective properties that was demonstrated by several *in vitro* and *in vivo* studies using models of ischemia, cerebral malaria, Alzheimer’s Disease (AD), Huntington’s Disease (HD), MS and Parkinson’s Disease (PD) (Martín-Moreno et al., 2011; Fernández-Ruiz et al., 2013; Mori et al., 2017; da Silva et al., 2018).

CBD reduced tau protein hyperphosphorylation (Casarejos et al., 2013; Aso et al., 2016) and the production of interleukins and nitric oxide in the brain (Iuvone et al., 2009; Walther and Halpern, 2010; Aso et al., 2016). In *in vitro* models of AD and MS, CBD pretreatment reduced ROS accumulation, mitochondrial dysfunction, lipid peroxidation, caspase-3 levels and DNA fragmentation (Iuvone et al., 2004; Vallée et al., 2017).

CBD also promotes neuroprotective action in an animal model of PD, presumably because of their antioxidant properties (García-Arencibia et al., 2007). In PD’s animal model produced after the unilateral injection of 6-hydroxydopamine (6-OHDA) into the medial forebrain bundle, the administration of CBD immediately after the injury, recovered the dopamine depletion in nigrostriatal neurons, but did not revert the consequences of the dopaminergic neurodegeneration when the treatment started 1 week after the injury (Noor et al., 2002; García-Arencibia et al., 2007).

An open pilot study conducted in PD patients showed that CBD, when associated with medications used in the clinic to treat PD, reduced psychotic symptoms without influencing the cognitive and motor signs of the disease (Zuardi et al., 2009). In a subsequent clinical trial, Chagas and colleagues suggested that CBD may improve motor symptoms, sleep disturbances and, the quality of life in patients with PD (Chagas et al., 2014).

The neuroprotective effects of CBD have also been described in MS. In a mouse model of MS, CBD administration mitigated experimental autoimmune encephalomyelitis (EAE) by increasing anti-inflammatory and reducing pro-inflammatory cytokines (Elliott et al., 2018). In patients with MS, oromucous spray composed of Δ^9^-THC/CBD (Sativex^®^) promoted a reduction of spasticity without serious adverse effects (Collin et al., 2010; Notcutt et al., 2012). The mechanism of action of Sativex in humans is not well elucidated, however in animal models of EAE, the treatment with Sativex-like combination of Δ^9^-THC and cannabidiol attenuated the progression of EAE through the activation of CB_1_ receptors (Hilliard et al.,  2012; Moreno-Martet et al., 2015). Currently, Sativex® is approved in some countries for the treatment of MS-related spasticity and neuropathic pain (Fraguas-Sánchez and Torres-Suárez, 2018).

Similar to PD, HD is characterized by changes in behavior and motor disorders (Niccolini, 2014). In HD animal model, the administration of CBD completely reversed 3-nitro propionic acid (3NP) reductions in mRNA levels for SOD-2. However, a trial conducted in patients with HD, Sativex® did not significatively improve motor, cognitive or psychiatric impairment related to HD (López-Sendón Moreno et al., 2016).

CBD can also exert neuroprotective effects in animal models of brain ischemia. In brain slices of newborn rats submitted to oxygen and glucose deprivation CBD reduced acute (LDH efflux to the incubation medium) and apoptotic (caspase-9 concentration in tissue) (Castillo et al., 2010). CBD prevented the increase of excitotoxicity, oxidative stress and inflammation in hypoxic-ischemic (HI) brain injury model in newborn pigs. Mori et al. (2017) demonstrated that short-term treatment with CBD results in global functional recovery in ischemic mice. The main mechanisms of neuroprotection are mediated by the reduction of oxidative stress and anti-inflammatory action induced by CBD treatment.

## The Widespread Functions of Glial Cells in the Brain

The first records of the use of the term glia (from the ancient Greek: glue) in the field of neuroscience are from 1850 (as a reference for their former attributed function: put the SNC together). Rudolf Virchow proposed the term neuroglia to describe the “substance … which lies between the proper nervous parts, holds them together and gives the whole its form in a greater or lesser degree.” The term was also generally used to emphasize the systematic identification of glial cells associated with pathological changes, such as glial tumors, encephalitis, and myelitis (Virchow, 1856; Fan and Agid, 2018). Later, several neuroanatomists and neurophysiologists have characterized different cell types as part of neuroglia. These groups of cells were divided into two categories according to their embryonic origin: macroglia (of ectodermal origin: astrocytes, oligodendrocytes, and polydendrocytes) and microglia (originated from the yok Salk’s).

Astrocytes (from the Greek *Astron*: star, while *kytos*: hollow vessel) were the first to be identified by Michael Von Lenhossék in 1891 (Von Lenhossék, 1895). The most numerous cells in the CNS, astrocytes play a crucial role in its metabolic support, maintenance of ionic and osmotic homeostasis, regulation of neurotransmitter levels in the synaptic cleft, control of the communication between the brain and the periphery, support for synaptic signaling and mediation of neurovascular coupling. They also actively participate in the formation, maintenance, and proper signaling of the synapses (Khakh and Sofroniew, 2015; Kozela et al., 2017).

Astrocytes are classified in subtypes based on their morphological and functional properties: protoplasmic, fibrous, interlaminar, and varicose projection. The last two subtypes listed are found in primates’ brains, but not in rodents (Tabata, 2015). Protoplasmic astrocytes are widely distributed in the gray matter associated with neuronal synaptic terminals to comprise the tripartite synapses (Kozela et al., 2017; Tabata, 2015). On the other hand, fibrous astrocytes are primarily located in the white matter and express higher levels of Glial fibrillary acidic protein (GFAP), a protein described by Ramon y Cajal in the early 1900s as a marker of astrocytes. Nevertheless, expression of GFAP varies depending on the brain region and, in healthy states, some astrocytes do not express GFAP (Khakh and Sofroniew, 2015). Other molecules used as astrocytic markers are GLAST, GLT-1, connexin 30, S100β, glutamine synthetase, aquaporin four, and aldehyde dehydrogenase one family, member L1 (Yang et al., 2011; Khakh and Sofroniew, 2015).

Oligodendrocytes were described for the first time by Ford Robertson in 1899 and called mesoglia. Later, Cajal and his student Pio del Río-Hortega designated these cells as oligodendroglia (the interfascicular glia) (Ramóny Cajal, 1920). This glial cell is responsible for myelin production, providing energy-efficiency to neurons and maintaining axonal integrity through trophic and metabolic support (Michalski and Kothary, 2015; Simons and Nave, 2016).

Among the glial cells, microglia had the most intriguing discovery. In 1841, Gluge described phagocytic cells in the damaged brain for the first time. He called these cells “inflammatory corpuscles.” Microglia was later described and named by other neuroanatomists: foam cells (Virchow, 1856), road cells (Nissl, 1899), granuloadipose cells (Achúcarro, 1909) and scavenger cells (Merzbacher, 1910). Although called by many names, they were always described as phagocytic cells in damaged or inflamed brain tissue (Rezaie et al., 2014). Finally, the term microglia was minted by the fantastic work of Pio Del Río-Hortega in 1919 to refer to these cells that are the resident macrophages of the CNS and promote early host defense against infections or injuries (Tremblay et al., 2015).

In the 1980s, the fourth known type of glial cells, distinct from mature oligodendrocytes, astrocytes, and microglia, the polydendrocytes (also called NG2 cells, oligodendrocyte precursor cells or synaptocytes), was described. These cells express the chondroitin sulfate proteoglycan NG2 and are present in both the gray and the white matter (Dawson et al., 2003; Hughes et al., 2013). Different from other glial cells, polydendrocytes are considered bipotential cells that putative generate both oligodendrocytes and protoplasmic astrocytes (Nishiyama et al., 2009). In several animal models of demyelination, NG2 cells are shown to rapidly replace oligodendrocytes (Gensert and Goldman, 1997; Abílio and Reynolds, 1999; Tanaka et al., 2004), suggesting that they play a role in remyelination and brain homeostasis. Nonetheless, their function goes further than the generation of new oligodendrocytes in the brain. NG2 cells are in close proximity to neurons and may be an integral component of synaptic connections (Butt et al., 2005).

After more than 150 years of research, the heterogeneous population of glial cells is much more than structures that fill the empty spaces between neurons. They play essential roles for the maintenance of critical aspects of brain homeostasis, including: (1) energy metabolism; (2) ion homeostasis; (3) network and cellular homeostasis; (4) neurotransmitter clearance; (5) organ homeostasis and osmotic control; and (6) immune response (for review see, Jäkel and Dimou, 2017).

## Glial Cells and Their Response to CNS Damage

Reactive gliosis is observed under various neurological conditions such as infection, ischemia, trauma, and neurodegeneration, and psychiatric disorders. The activation of these glial cells usually involves hypertrophy and proliferation, changes in the patterns of gene expression, and release of chemokines, cytokines, and neurotrophic factors. Once released, these factors can either induce neuroprotection or produce damage in the neural tissue (Lee and Chung, 2019).

Activation of microglia is a common hallmark of a diverse range of neurodegenerative diseases, including AD (Esposito et al., 2007, 2011), MS, PD, HD (Sapp et al., 2001), and is considered to be responsible for the ongoing inflammatory condition occurring in neurodegenerative diseases. Of note, the activation of glial cells during insults or neuropsychiatric conditions are not only a secondary response to damage, but could also play a pathophysiological role in its development (Jonsson et al., 2013; Hong et al., 2016; Sekar et al., 2016).

For instance, reactive astrocytes and microglia are found to be important for protein misfolding removal in neurodegenerative diseases. Activated glial cells can be found around Aβ plaques and play beneficial or harmful roles in disease progression (Nagele et al., 2003; Olabarria et al., 2010; Simpson et al., 2010). A similar panorama is found in PD brains, were reactive astrocytes and microglial cells can be found close to *a*-synuclein inclusions. Astrocytes can internalize Aβ and *a*-synuclein *in vitro* (Wakabayashi et al., 2000; Wyss-Coray et al., 2003; Pihlaja et al., 2008; Lee et al., 2010; Braidy et al., 2013). Like astrocytes, microglial cells are located around Aβ plaques and *a*-synuclein inclusions in both human and mouse brains (Perlmutter et al., 1990; Bolmont et al., 2008; Grathwohl et al., 2009).

Oxidative stress and pro-inflammatory mechanisms are actively involved in misfolding protein aggregation. On the other hand, misfolded proteins can also lead to excessive oxidative stress and inflammation leading to neurotoxicity and neurodegeneration. Glial cells not only can produce ROS and proinflammatory signals, but also suffer the consequences of this “unfriendly” neurodegenerative environment (Singh et al., 2019). In addition, glial cells are involved in maintaining the inflammatory state during epilepsy by the release of inflammatory cytokines (Ravizza et al., 2008; Devinsky et al., 2013; Do Val-da Silva et al., 2017).

In the case of psychiatric disorders, the “new” Neuro-immune hypothesis states that circulating levels of cytokines and immune cells are found increased in patients with mood disorders, schizophrenia, and post-traumatic stress disorder. However, glial reductions are described by independent laboratories in different brain areas. For instance, mixed data is found in this regard in the anterior cingulate cortex, prefrontal cortex, and orbitofrontal cortex of patients with mood disorders (Stockmeier and Rajkowska, 2004; Wilczyńska et al., 2018). Similarly, in schizophrenia, some studies demonstrate increased microglia activation. In contrast, others failed to replicate earlier studies and found no differences between patients and healthy controls (Trépanier et al., 2016). Therefore, although the role of gliosis in neurodegenerative diseases is established, in the case of psychiatric disorders, the panorama is not so clear. However, it is important to remember that besides their vital functions in the homeostatic and pathological brain, glial cells express several pharmacological receptors that are used as primary targets by drugs, including CBD, to produce their therapeutic actions, as we will discuss in the next topics.

## CBD Effects That Not Include Glial Cells in Its Mechanism of Action (Yet)

Why it has been so difficult to figure out the mechanism of action of CBD? In every storyline, to understand the entire plot, it is essential to know the characters. Are all potential characters being considered to construct CBD’s storyline? Most studies aimed at investigating these mechanisms do so with the assumption that they are located in neurons. Even when putative brain sites of CBD action are identified, their precise cellular location is unknown (Khan et al., 2020). Khan et al. (2020) showed that CBD exerted a CB_1_-dependent panicolytic-like effect after the pharmacological excitation of the ventromedial hypothalamus in rats. Neuronal excitatory activity has been shown to cause an accumulation of lactate. Brain regions such as the hypothalamus respond to pH changes caused by the activity-dependent increase in lactate (Maddock et al., 2013). Lactate is generated via glycolytic metabolism, mainly in astrocytes, and then transferred to neurons as an energy source (Riske et al., 2017). Recently, it has been shown that mitochondrial CB_1_ receptors present in astrocytes dampen lactate production (Jimenez-Blasco et al., 2020). Therefore, activation of astrocytic CB_1_ could diminish activity-dependent lactate increase and interfere in neural activity and in resulting behavioral responses.

Other CBD effects that depend on cannabinoid CB_1_ and CB_2_ receptors, including the disruption of fear memories, alterations in reward-related responses, and neuroprotection (Castillo et al., 2010; Pazos et al., 2013; Stern et al., 2017; Bi et al., 2020; Galaj et al., 2020; Raymundi et al., 2020), could also involve glial cells. CB_1_ receptors, apart from being expressed in astrocytes, are also found in microglia, oligodendrocytes, and NG2 cells (Molina-Holgado et al., 2002; Cabral, 2005; Stella, 2010). The cellular location of CB_1_ receptors in the CNS is relevant for determining its signaling route. While in neurons, CB_1_ receptors activate G_i_ and decrease neurotransmitter release from pre-synaptic terminals, in astrocytes these receptors increase intracellular calcium concentrations and ultimately lead to a potentiation of synaptic transmission (Navarrete and Araque, 2010). CB_2_ receptors have also been found in astrocytes, oligodendrocytes, and microglia. There is a discussion, however, whether in these cells, CB_2_ receptors are expressed in physiological conditions or only induced in pathological conditions (Molina-Holgado et al., 2002; Stella, 2010; Cassano et al., 2017; Tanaka et al., 2020).

Microglia show remarkable plasticity and can adopt a spectrum of polarized states in response to microenvironmental cues. Interestingly, cannabinoid receptors expressed in these cells vary depending on their activation profile. In intact, healthy brain tissue, microglial cells behave predominantly in a resting state, a condition where immunostaining assays often do not find CB_1_ positive cells (Stella, 2010). Regarding CB_2_ receptors, studies performed in rodent or human samples in general reports the lack of CB_2_ receptors or their presence in levels too low to be quantified (Munro et al., 1993; Galiègue et al., 1995; Schatz et al., 1997; McCoy et al., 1999). On the other hand, as shown in mice models and patients, during specific neuroinflammatory conditions CB_2_ receptor is upregulated in activated microglia. This effect has been associated with responses to microenvironment changes such as the presence of pathogens, cytokines, and other molecules (Maresz et al., 2005; Palazuelos et al., 2009). The regulatory mechanisms that drive the expression of specific microglia phenotype and whether CBD exert its anti-inflammatory effects by modulating these mechanisms remains to be understood. Mecha et al. (2015) reported that AEA and 2-arachidonoylglycerol are independently regulated in microglia by specific anti-inflammatory cues, suggesting that endocannabinoid signaling plays crucial role in regulating microglia phenotype during neuroinflammatory and neurodegenerative conditions.

The enhancement of endocannabinoid signaling, specially AEA, has been proposed as one of the actions of CBD in the CNS (Watanabe et al., 1996; Bisogno et al., 2001; Campos et al., 2013a). Neurons are not the only cells to populate the CNS that produce and release endocannabinoids. Microglial cells and astrocytes also express the AEA-synthesizing enzyme NAPE-PLD (Kallendrusch et al., 2012), and it was shown that *in vitro* astrocytes can produce AEA (Walter et al., 2002). Gabrielli et al. (2015) showed that microglial cells produce AEA and release the endocannabinoid to the perivascular space in association with macrovesicles or exosomes, modulating the activity of inhibitory neurons.

FAAH and fatty-acid binding proteins (FABPs), which participate in the transport and hydrolysis of AEA, respectively, are expressed in microglia, astrocytes, oligodendrocytes, and NG2-positive cells (Egertová et al., 2003; Kallendrusch et al., 2012; Graves, 2013; Sharifi et al., 2013; Young et al., 2013; Duffy et al., 2017; Gerstner et al., 2017; Foerster et al., 2020). FABP5 and FABP7 have been shown to regulate the proliferation of NG2-positive cells and their differentiation to oligodendrocytes (Sharifi et al., 2013). Brains astrocytes expressing FABP7 are largely concentrated at the hippocampal neurogenic niche, often in close proximity to proliferating precursor cells located in subgranular zone of the dentate gyrus (DG) (Boneva et al., 2011; Young et al., 2013). CBD has important effects in this neurogenic niche, and local enhancement of AEA levels is one of the mechanisms associated with its pro-neurogenic actions (Campos et al., 2013a; Fogaça et al., 2018).

Glial cells are important for the process of adult hippocampal neurogenesis. In the neurogenic niches, apoptosis in an important mechanism. Microglial cells located in subgranular zone of the DG are sensors of cell death and rapidly eliminate cell debris through phagocytosis, an essential step of the neurogenic process (Sierra et al., 2010; Diaz-Aparicio et al., 2020). In turn, astrocytes control the proliferation, survival, and differentiation of progenitor cells (Song et al., 2002; Barkho et al., 2006; Lu and Kipnis, 2010; Terrillion et al., 2017; Wilhelmsson et al., 2019; Asrican et al., 2020). This property seems to be restricted to astrocytes localized at neurogenic niches, indicating that these cells might provide regionally-specific signals that allow certain brain areas to maintain its capability of generating new cells (Song et al., 2002). Besides, Sultan et al. (2015) demonstrated that astrocytes are essential for regulating the survival and integration of newly-born neurons into the adult hippocampal synaptic circuitry. CBD has been shown to increase the proliferation, survival, differentiation, and migration of precursor cells in the subgranular zone of the dentate gyrus of the hippocampus (Esposito et al., 2011; Campos et al., 2013a; Schiavon et al., 2016; Fogaça et al., 2018; Luján et al., 2018). Nevertheless, it is still unknown whether CBD acts directly at progenitor cells and neuroblasts or indirectly by modulating the function of local cells that control the neurogenic process.

Neural stem cells located in the neurogenic niches of the adult brain are multipotent and can give rise to astrocytes, even though the generation of new-astrocytes in the subgranular zone is usually underestimated and very rarely evaluated. In fact, Bonaguidi et al. (2011), using a genetic non-invasive approach to evaluate the lineage tracing of nestin-positive radial glia-like precursors and showed that the number of newly-born astrocytes is similar to that of new neurons in the adult dentate gyrus. Nevertheless, none of the studies that evaluated CBD effects in the subgranular zone of the dentate gyrus addressed astrogliogenesis. Some works used as the sole measure of neurogenesis the number of cells that express doublecortin (DCX) in the dentate gyrus (Esposito et al., 2011; Mori et al., 2017). Even though DCX is widely proposed as a marker of cells compromised with the neuronal phenotype, it has been shown that glial cells, especially some polydendrocytes, can also express DCX (Boulanger and Messier, 2017). Besides, when the fate of newly born cells was evaluated, only markers of the neuronal phenotype were used (Campos et al., 2013a; Fogaça et al., 2018; Luján et al., 2018).

The potential impact of CBD on astrogliogenesis in the dentate gyrus remains unknown. Campos et al. (2013a) showed that two weeks of treatment with CBD (30 mg/kg) increased the proliferation of precursor cells in the dentate gyrus of wild-type animals. The drug, however, failed to change this proliferation in the hippocampus of ganciclovir-treated mice expressing the thymidine kinase (TK) under the control of the GFAP promoter (GFAP-TK). The dampened pro-proliferative effect of CBD prevented its anxiolytic-like effect in chronically stressed GFAP-TK mice. The study concluded that an intact adult hippocampal neurogenesis capacity is needed for the anxiolytic response generated by CBD in animals exposed to chronic stress. Nonetheless, in transgenic GFAP-TK mice treated with ganciclovir there is a depletion of radial glia-like GFAP-positive precursor cells, which could potentially impact not only neurogenesis but also the astrogliogenesis in the dentate gyrus. Whether or not astrogliogenesis could play a role in the anxiolytic-like response triggered by CBD in chronically stressed mice, remains to be investigated.

Another approach used to address the relevance of neurogenesis in CBD effects is the pharmacological inhibition of cell division. Luján et al. (2020) showed that a 10-days treatment with CBD (20 mg/kg) reduces cocaine self-administration, and this effect was blocked in mice previously treated systemically with the chemotherapy drug temozolomide. The study proposed that the alkylating agent would affect the DNA replication and mitosis of cells with low proliferative profile, like neural precursor cells. Indeed, the chemotherapy drug reduced the number of new neurons generated in the dentate gyrus after CBD treatment. The authors concluded that adult neurogenesis could be essential for the reduction of cocaine intake induced by CBD. Again, this study does not address astrogliogenesis. Besides, outside neurogenic niches of the adult brain, polydendrocytes (NG2 cells) are the CNS main proliferative cells (Geha et al., 2010). These cells, apart from being oligodendrocytes precursors, control ion homeostasis, remyelination, receive synaptic inputs from glutamatergic and GABAergic neurons, and might even be able to differentiate into neurons (Nishiyama et al., 2009; Nishiyama et al., 2014). A pharmacological protocol that interferes with cell division could affect the proliferation and function of NG2 cells. No study so far, nonetheless, has addressed the role of NG2 cells on the effects of CBD.

Stress is as a common risk factor for most of the psychiatric disorders for which CBD is proposed to be effective. In animal models, repeated CBD treatment counteracts the effects of chronic stress exposure. Fogaça et al. (2018) demonstrated that two weeks of treatment with CBD (30 mg/kg) prevented stress-induced impairment in synaptic plasticity, represented by a decrease in dendritic arborization and the number of dendritic spines density in granular neurons of the dentate gyrus. Chronic stress alters glial function and, just like CBD, pharmacological or genetic targeting these stress-induced changes modify its behavioral and neuroplastic consequences. Chronic stress increases astrocyte number and microglial activation in the dentate gyrus of the hippocampus (Machado-Santos et al., 2019; Du Preez et al., 2020). Yu et al. (2019) showed that chronic stress decreases the expression of the astroglial glutamate transporter-1 (GLT-1) in the hippocampus after ischemic stroke, which was accompanied by impaired synaptic plasticity and depressive-like behavior. Ceftriaxone, an antibiotic known to increase GLT-1 expression, counteracted the deleterious behavioral and neuroplastic effects of stress exposure (Yu et al., 2019). Besides, Hao et al. (2020) demonstrated that astrocyte chemogenetic inhibition in the hippocampus and prefrontal cortex, and microglial depletion reverse the behavioral consequences of a ten-day exposure to social defeat stress.

The brain-derived neurotrophic factor (BDNF) has been implicated in several brain functions. Decreased levels of BDNF is commonly associated with stress-related disorders, including depression and anxiety. BDNF modulates neuronal as well as glial functions. Ye et al. (2011) showed that the intrahippocampal infusion of BDNF restored the levels of the astrocytic proteins GFAP and S100b in stressed rats. Moreover, BDNF overexpression in hippocampal astrocytes increased neurogenesis and induced an anxiolytic-like response in the novelty suppressed feeding test (Quesseveur et al., 2013). Besides, the antidepressant fluoxetine has been shown to induce an ATP-mediated increase in BDNF in hippocampal astrocytes. This ATP-dependent mechanism is directly related to the antidepressant-like effect triggered by this drug (Kinoshita et al., 2018). CBD also seems to affect BDNF levels, although the cell types involved in this effect are unknown. Sales et al. (2018) showed that acute CBD treatment induces a rapid antidepressant-like effect accompanied by an increase in BDNF levels in the hippocampus and prefrontal cortex. CBD antidepressant-like effect was blocked by the intracerebroventricular administration of K252a, an antagonist of BDNF receptor TrkB. Furthermore, chronic CBD treatment attenuates the decreases in BDNF and the astroglial protein GFAP observed in the hippocampus of diabetic animals submitted to a model of chronic cerebral hypoperfusion (Santiago et al., 2019).

As discussed before, the serotonergic system is also frequently associated with CBD effects. Treatment with this drug increased serotonin levels in the prefrontal cortex (Linge et al., 2016). The pretreatment with an inhibitor of serotonin synthesis abolished the antidepressant-like effect of acute CBD treatment (Sales et al., 2018). This increase in serotonin levels induced by CBD has been attributed to its action at serotonergic 5-HT1A receptors, once CBD acts as an agonist of 5-HT1A receptors (Russo et al., 2005). CBD action at 5-HT1A receptors, however, could be also indirect, by acting as an allosteric modulator (Rock et al., 2012).

Apart from its antidepressant-like properties, other actions of CBD seem to depend on 5HT1A receptors. Administration of WAY100635, a 5-HT1A antagonist, prevented the antipsychotic-like effects of CBD in a mouse model of schizophrenia based on chronic NMDA receptor antagonism (Rodrigues-da-Silva et al., 2020). Serotonin-dependent synaptic plasticity might depend on 5-HT1A glial receptors, once serotonin modulates the density of synaptic connections in the dentate gyrus of the hippocampus via astroglial 5-HT1A receptors (Wilson et al., 1998). Also, glial cells are able to modulate extracellular serotonin levels by expressing the serotonin transporter (Inazu et al., 2001). Other serotonin receptors might modulate astrocytic calcium signaling (Schipke et al., 2011) and microglial exosome release (Glebov et al., 2015).

In addition to its interaction with membrane-associated receptors and related downstream signaling cascades, CBD can also bind to nuclear receptors. Converging evidence obtained over the last decade indicate that the peroxisome proliferator-activated receptor-γ (PPAR-γ) is a nuclear target to CBD. This receptor modulates the expression of genes related to the control of central and peripheral inflammation and immune responses (Bensinger and Tontonoz 2008; Wang et al., 2013). Activation of this receptor by CBD could interfere with transcriptional pathways responsible for inflammatory responses, eg, modulation of NF-κB signaling (Esposito et al., 2007). Therefore, PPAR-γ is frequently associated with CBD neuroimmune effects. This receptor is expressed by astrocytes, microglial cells, oligodendrocytes, NG2 cells, and neurons (Bernardo et al., 2000; Cristiano et al., 2001; Gray et al., 2012; Ke et al., 2014; Ding et al., 2020).

CBD might also exert its protective effects by reducing the permeability of the blood-brain barrier (BBB). Mecha et al. (2013) showed that in animal model of MS, CBD neuroprotective effects were mediated by adenosine A2, another receptor frequently linked to its neuroimmune modulatory action (Castillo et al., 2010; Mecha et al., 2013).

**FIGURE 1:**
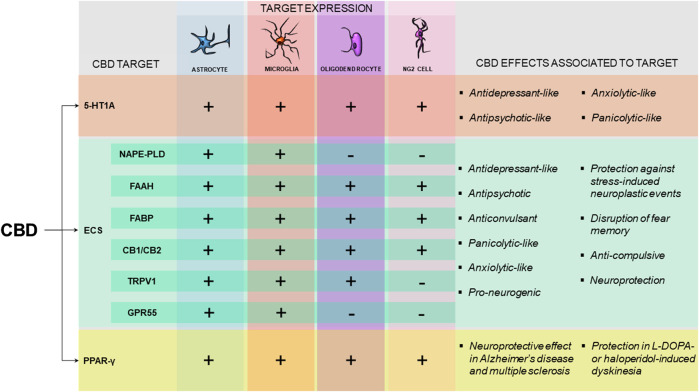
Pattern of expression and effects associated with the molecular targets potentially involved in CBD mechanism of action in glial cells. Schematic representation pointing the expression of 5-HT1A receptor, proteins of the endocannabinoid system (ECS) and the PPAR-γ receptor in astrocytes, microglia, oligodendrocytes and NG2 cells (Azmitia et al., 1996; Bernardo et al., 2000; Egertová et al., 2003; Zhang et al., 2011; Gonzalez-Reyes et al., 2013; Graves, 2013; Sharifi et al., 2013; Young et al., 2013; Ke et al., 2014; Fan and Agid, 2018; Duffy et al., 2017; Viudez-Martínez et al., 2018; Kong et al., 2019; Yang et al., 2019; Ding et al., 2020) (+) indicates that there is evidence that the protein has been found in the specific cell type (−) indicates the absence of evidence for the expression of the molecular target in the specific cell type. For the ECS, NAPE-PLD, FAAH and FABP are considered as the potential targets involved in the increased AEA availability induced by CBD treatment. The effects of CBD that have been shown to be dependent on 5-HT1A (Campos and Guimarães, 2008; Zanelati et al., 2010; Campos et al., 2013a; Rodrigues da Silva et al., 2020), on the ECS (Casarotto et al., 2010; Leweke et al., 2012; Stern et al., 2012; Campos et al., 2013b; Pazos et al., 2013; Sartim et al., 2016; Fogaça et al., 2018; Khan et al., 2020) or on PPAR-γ receptors (Esposito et al., 2011; dos-Santos-Pereira et al., 2016; Giacoppo et al., 2017; Sonego et al., 2018) are highlighted. *CBD*, Cannabidiol; *5-HT1A*, 5-hydroxytryptophan 1 A receptor; *ECS*, endocannabinoid system; *NAPE-PLD*, N-acyl phosphatidylethanolamine-specific phospholipase D; *FAAH*, fatty-acid amide hydrolase; *FABP*, fatty acid binding protein; *CB1/CB2*, cannabinoid receptor one/cananbinoid receptor two; *TRPV1*, transient receptor potential vanilloid one; *GPR55*, G coupled receptor 55; PPAR-γ, Peroxisome proliferator-activated receptor-γ.

Glial cells respond to neuronal stimulation, releasing gliotransmitters (like glutamate, prostaglandins, and ATP) and actively affecting neuronal firing rate and synaptic plasticity in the developing and adult brain (Haydon, 2001). The description of the tripartite synapse, a concept that could even be expanded for a quadpartite or even a pentapartite synapse, highlighted that the normal brain requires proper functioning of glial cells that ultimately maintain the homeostasis of the system (Perea et al., 2009; Schafer et al., 2013; De Luca et al., 2020). This new perspective also impacts how psychopharmacology looks at the action of psychoactive drugs like CBD. Investigating the non-neuronal cells involved in the actions of CBD might be as relevant as identifying the receptors targeted by this phytocannabinoid. As discussed above, several of the receptors target by CBD are present in astrocytes, microglial cells, oligodendrocytes, and NG2 cells. Very few studies, however, have investigated whether glial cells play a role in CBD potential therapeutic effects. Figure 1 summarizes the main receptors and enzymes present in glia that have been shown to participate in CBD actions.

## Current Evidence Showing That CBD Acts by Modulating Glial Cells Functions

### CBD and Astrocytes

In studies evaluating CBD effects in astrocytes, GFAP and S100β are the most commonly used astroglial markers. di Giacomo et al. (2020) showed that astrocytes treated for 48 h with 1 μM of CBD present increased cell proliferation. The same concentration of CBD protected astrocytes from oxidative damage and apoptosis after exposure to hydrogen peroxide *in vitro*. In human astrocytes in co-culture with human brain microvascular endothelial cells submitted to oxygen-glucose deprivation, CBD increased cell survival, evidenced by a reduced lactate dehydrogenase (LDH) release and decreased VCAM-1 expression (Hind et al., 2016). In human astrocytes in monoculture, however, CBD increased cell damage levels, with increased LDH levels, at the concentration of 10 μM (Hind et al., 2016). Auzmendi et al. (2020) used a primary astrocyte culture to show that CBD treatment, in a concentration-dependent manner, inhibits the active efflux of the P-glycoprotein substrate rhodamine-123.

Lafuente et al. (2011) showed that a hypoxic-ischemic lesion in newborn piglets led to a decrease in cortical GFAP-positive cells and an increase in the levels of S100β in the cerebrospinal fluid, indicating astrocytic damage. CBD treatment attenuated the alterations in astrocytic markers, indicating that it protects astrocytes from ischemic injury. Mori et al. (2017) showed that 21 days after bilateral carotid artery occlusion in adult mice, there was an increase in immunoreactivity for GFAP in the CA1 and CA3 hippocampal regions, with augmented total levels of GFAP in the hippocampus. CBD prevented these alterations. Furthermore, in newborn rats exposed to collagenase-induced germinal matrix hemorrhage, CBD treatment reduced the number of reactive astrocytes (GFAP-positive) and caspase-3 positive-astrocytes in the perilesional area (Abrantes de Lacerda Almeida et al., 2019).

Esposito et al. (2011) showed that in cultured astrocytes, CBD treatment decreased the *ß*-amyloid-induced release of pro-inflammatory mediators such as nitric oxide, TNF-α, S100B, and IL-1β. CBD effects were abolished by the PPAR-γ antagonist, GW9662. *In vivo* data also showed that CBD, via a PPAR-γ-dependent mechanism, diminished the pro-inflammatory response triggered by the intrahippocampal injection of *ß*-amyloid (Esposito et al., 2011). Through this PPAR-γ-mediated action, CBD is proposed to reduce neuroinflammation and protect neurons from neurodegeneration in AD (Valée et al., 2017). Also, CBD systemic administration dose-dependently reduced the increased hippocampal levels of GFAP mRNA and S100β caused by local injection of *ß*-amyloid (Esposito et al., 2007, 2011).

Hind et al. (2016) showed in an *in vitro* model using a co-culture of human brain microvascular endothelial cells and astrocytes that CBD decreased BBB permeability via PPAR-γ-dependent mechanism. They propose that this mechanism contributes to the protective effects of CBD in ischemic stroke.

In a rat model of epilepsy based on the chronic treatment with the GABAergic antagonist pentylenetetrazol, co-treatment with CBD prevented the increase in the number of GFAP-positive cells in the CA1 and CA3 hippocampal areas (Mao et al., 2015). Similarly, Gomes et al. (2015) showed that repeated CBD treatment attenuated the increased GFAP-positive cell number in the medial prefrontal cortex in a mouse model of schizophrenia.

Interestingly, although the studies described so far focused on brain glial cells, enteric astrocytes might also be affected by CBD treatment. De Filippis et al. (2011) showed that LPS administration in mice increased intestinal S100β, an effect blocked by CBD. Moreover, CBD attenuate increased S100β levels observed in cultures generated from intestinal biopsies obtained from patients with ulcerative colitis (De Filippis et al., 2011).

### CBD and Oligodendroglia

Oligodendrocytes have been associated with white matter dysfunction in neurodegenerative and psychiatric disorders such as schizophrenia (Hof et al., 2002; Flynn et al., 2003).

In this sense, cannabinoids counteract demyelination in some conditions (Molina-Holgado et al., 2002; Mecha et al., 2013; Tomas-Roig et al., 2015). Therefore, CBD and analogs might represent a useful neuroprotective candidate to manage neuropsychiatric white matter-associated deficits. Several lines of evidence highlight CBD benefits toward glial damage in diverse models ranging from pediatric conditions, such as demyelination induced by neonatal hypoxia (Ceprián et al., 2017), to age-related diseases, such as PD and AD, for instance (Benito et al., 2003; García-Arencibia et al., 2009; Fernandez-Ruíz, 2019). In spite of the remarkable therapeutic potential of CBD to demyelinating diseases, more studies are needed to produce a deep understanding of the machinery involved in CBD anti-inflammatory and antioxidant mechanisms are still unknown.

Mecha and colleagues (2012) reported anti-inflammatory effects of CBD (1uM) in OPCs in an independent manner of CB_1_, CB_2_, TRPV1, and PPAR-γ. This effect was intracellularly mediated by a decrease in the phosphorylation of proteins that coordinates endoplasmic reticulum apoptotic pathway, the RNA-activated serine/threonine kinase (PKR) and translation initiation factor 2α (eIF2α) (Mecha et al., 2012). However, in adult oligodendrocytes derived from the rat optic nerve, CBD (1uL) promoted disruption of mitochondrial membrane potential along with elevation of intracellular calcium and increase of ROS production. This effect leads to a decrease in oligodendrocyte viability via a mechanism of CB_1_, CB_2_, and TRPV1, but mediated by the activation of both caspase-dependent and independent cell death pathways (Mato et al., 2010). Considering that both studies have used the same concentration of CBD, the divergences in CBD effects might be related to differences in the stage of cells maturation (OPCs *vs.* mature oligodendrocytes). Ceprián et al. (2017) first reported maturation stage-dependency of CBD effects in oligodendrocytes. In the ipsilateral cortex, but not in the white matter, CBD restored mature oligodendrocyte cell density after hypoxic brain injury. In the white matter, CBD protected the axons, preserving appropriate myelination after injury (Ceprián et al., 2017). The authors conclude that the differences between CBD effects in the white matter and ipsilateral cortex could be explained by the distinct maturational stages of oligodendrocytes in these areas. Maturation from OPCs to immature oligodendrocytes in the white matter occurs first than in the ipsilateral cortex (Ceprián et al., 2019).

### CBD and Microglial Cells

The hypothesis of activated microglia as a key feature in neurodegenerative diseases and possibly in psychiatric disorders suggests that these cells may represent a new therapeutic approach.

Many lines of evidence suggest that cannabinoids are neuroprotective by promoting anti-inflammatory mechanisms. The effects of CBD have been related to the control of microglial migration, microglia activation, and the toxicity exerted by these cells by producing pro-inflammatory mediators (Rahimi et al., 2015)

A study by Hayakawa et al. (2008) demonstrated that the administration of CBD (3 mg/kg, i.p) immediately before and 3 h after cerebral artery occlusion prevented glial activation, as indicated by the reduction of Iba-1 expression in the infarcted area (Hayakawa et al., 2008). CBD treatment also diminished the infiltrate of immune cells such as neutrophils, macrophages, and monocytes, and decreased the infarct size in a CB_1_ and CB_2_ independent manner (Hayakawa et al., 2008).

The most straightforward association between CBD actions and microglial cells is the murine model of EAE, which resembles MS-like conditions. CBD ameliorated the disease progression while decreasing the activation of microglial cells in the spinal cord (Kozela et al., 2011). A decrease in microglial activation is also proposed as a possible mechanism of the reduced neuroinflammation and improved cognitive performance observed in mice submitted to a model of AD treated with CBD (Martín-Moreno et al., 2011; Watt et al., 2020).

Recent work by Sonego et al. (2018) has shown in a primary microglial culture that a PPAR-γ antagonist, GW9662, was able to block the protective effects of CBD on the enhancement of Iba-1 expression, the microglial production of ROS, and the NF-κB translocation to the nucleus induced by LPS (Sonego et al., 2018). The intracellular machinery responsible for CBD anti-inflammatory properties remains under investigation, although some mechanisms have been proposed. For instance, CBD was shown to be transported intracellularly by FABPs, which might explain the mechanism for nuclear receptors activation (Elmes et al., 2015). Moreover, CBD was also shown to be able to regulate inflammatory signaling of NF-κB by promoting inhibitory control of phosphorylation of specific kinases (eg p38 MA P kinase, PI3K), preventing the activation of pro-inflammatory genes (Esposito et al., 2007).

## Conclusions and Perspectives

Its wide range of putative therapeutic applications, safety profile, and still not very clear action mechanism makes CBD one of the most intriguing phytocannabinoid. Although several groups, including ours, have pointed to the involvement of different receptors (5-HT1A, CB1, CB2, PPAR-γ, Adenosine, TRPV1) and enzymes (FAAH, NAPE-PLD, enzymes related to oxidative stress process) in the effects of CBD, the contribution of specific neural cell types remains poorly understood.

In the present review we highlight the possibility that, in addition to neurons, glial changes could help to explain the complex pharmacology of CBD. Corroborating this proposal, acute or chronic administration of this drug can modify the expression of glial cell markers or induce changes in their morphology. On the other hand, *in vitro* studies using primary or immortalized culture of astrocytes and microglia have demonstrated that CBD can interfere with their function, especially during cell insults, such as inflammation (caused by LPS for instance). New studies using more sophisticated models (mini-brains, *in vivo* transgenic models) and aimed at observing the effects of CBD in the absence of specific glial cell responses (inhibition by Designer Receptors Exclusively Activated by Designer Drugs (DREADD)-based chemogenetics, or optogenetics) or their receptors (specific KO mice in glial cell populations) are needed to fully address this possibility.

It is unlikely, in our opinion, that CBD shares the same pharmacological mechanism in different brain disorders. Therefore, an important step to fully understand CBD mechanisms and potential role in the treatment of neuropsychiatric disorders is to unveil its interference in specific cellular subpopulations present in the CNS.

The complex pharmacokinetics of CBD poses another problem for its therapeutic use. Macrophages have been studied as possible alternatives for drug delivery (Jain et al., 2013). This opens the possibility of using microglial cells to delivery CBD to specific brain areas where their density would be more prominent due to pathological conditions.

**FIGURE 2:**
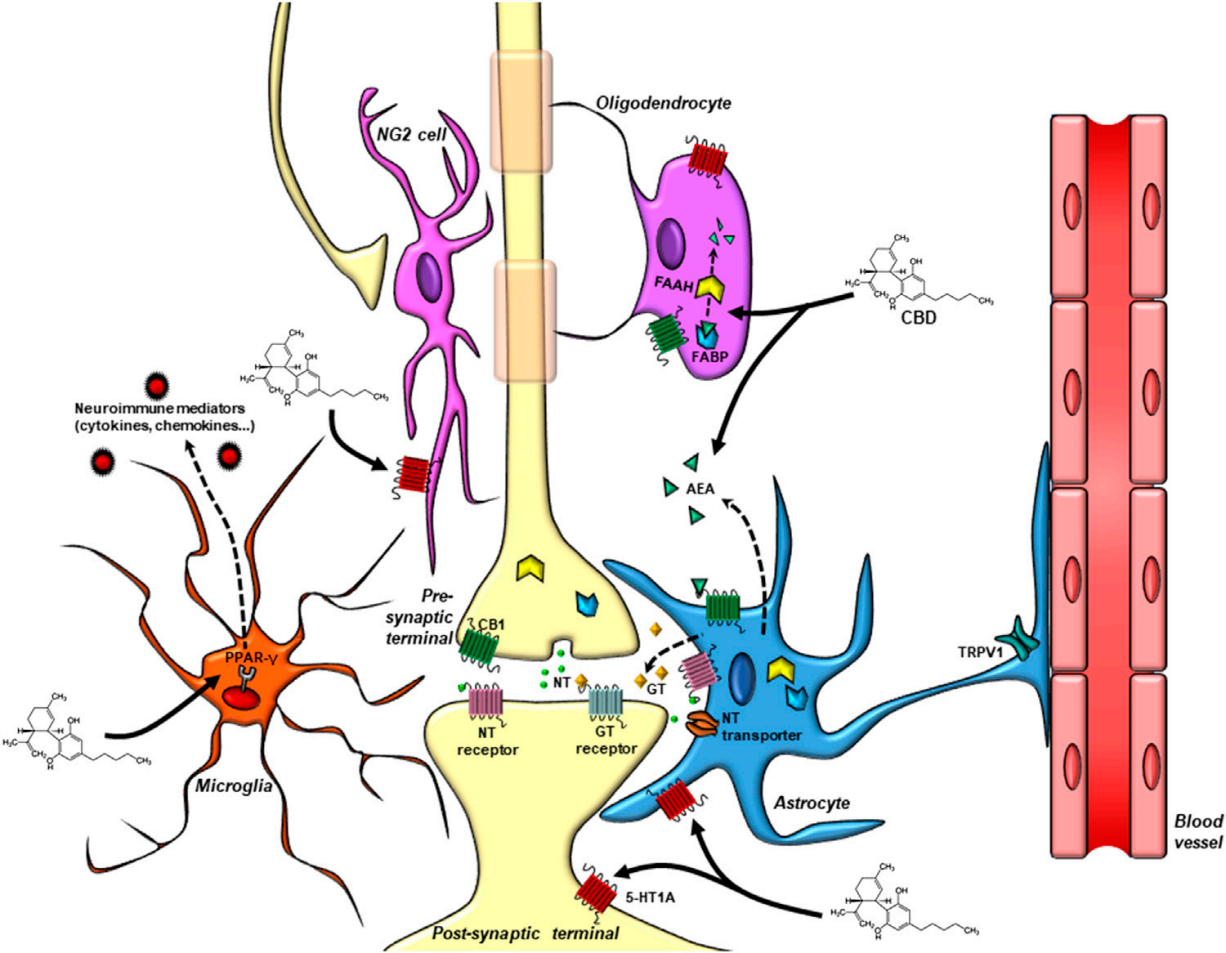
Drug targets for CBD in the “pentapartite” synapse. Glial cells display important roles in synaptic signaling and maintenance. They are in close proximity to the pre and postsynaptic terminals, express neurotransmitter receptors, control the content of neurotransmitters in the synaptic cleft via the expression of transporter proteins, and modulate synaptic activity by releasing gliotransmitters that act in neuronal receptors. Astrocytes also regulate the neurovascular coupling and the blood-brain barrier permeability. Microglial cells control the maintenance of synaptic connections, eliminate unwanted synaptic contacts and release neuroimmune modulators that regulate neuronal function. NG2 cells receive direct synaptic contacts from glutamatergic and GABAergic neurons. Oligodendrocytes maintain the myelin sheets necessary for proper impulse propagation in neurons. In this complex cellular dynamic, CBD targets are found not only in neurons, but also in glial cells. Astrocytes and microglial cells can synthetase and release AEA. Endocannabinoid levels are regulated by FABP and FAAH present both in neuronal and glial cells. Cannabinoid, TRPV1, PPAR-γ, and the serotonin 5-HT1A receptors, proposed to participate in CBD actions, are also present in glial cells and neurons. *CBD*, Cannabidiol; *NT*, neurotransmitter; *GT*, gliotransmitter; *AEA*, anandamide; *FAAH*, fatty-acid amide hydrolase; *FABP*, fatty acid binding protein; *CB*_*1*_, cannabinoid receptor one; *TRPV1*, transient receptor potential vanilloid 1; *5-HT1A*, 5-hydroxytryptophan 1 A receptor; PPAR-γ, Peroxisome proliferator-activated receptor-γ.

Therefore, the investigation of CBD effects on glial cells opens a new route of scientific opportunities. Understanding the role of “pentapartite” synapses (Figure 2) on CBD actions could be the “rosetta stone” to decipher the complexity behind its pharmacology.

## Author Contributions

All authors have approved this manuscript and have agreed to the Frontiers in Pharmacology's submission policies. We state that we are entirely responsible for the scientific content of the present work. We declare that this manuscript has not been published or is being considered for publication elsewhere.

## Funding

FFS is a FAPESP fellowship (2019/09178-3). MRA is a CAPES fellowship. This work is funded by FAPESP (2017/24304-0, 2015/0551-0, and 2019/25984-0). ACC is a level 2 CNPq productive Grant. FSG is a level 1A CNPq productive grant. All authors approved the final version of the manuscript.

## Conflict of Interest

The authors declare that the research was conducted in the absence of any commercial or financial relationships that could be construed as a potential conflict of interest.
